# A High-Sensitivity MEMS Accelerometer Using a Sc_0.8_Al_0.2_N-Based Four Beam Structure

**DOI:** 10.3390/mi14051069

**Published:** 2023-05-18

**Authors:** Zhenghu Zhang, Linwei Zhang, Zhipeng Wu, Yunfei Gao, Liang Lou

**Affiliations:** 1 School of Microelectronics, Shanghai University, Shanghai 201800, China; 2 The Shanghai Industrial μTechnology Research Institute, Shanghai 201899, China

**Keywords:** MEMS, piezoelectric accelerometers, ScAlN, sensitivity

## Abstract

In this paper, a high-sensitivity microelectromechanical system (MEMS) piezoelectric accelerometer based on a Scandium-doped Aluminum Nitride (ScAlN) thin film is proposed. The primary structure of this accelerometer is a silicon proof mass fixed by four piezoelectric cantilever beams. In order to enhance the sensitivity of the accelerometer, the Sc_0.2_Al_0.8_N piezoelectric film is used in the device. The transverse piezoelectric coefficient *d*_31_ of the Sc_0.2_Al_0.8_N piezoelectric film is measured by the cantilever beam method and found to be −4.7661 pC/N, which is approximately two to three times greater than that of a pure AlN film. To further enhance the sensitivity of the accelerometer, the top electrodes are divided into inner and outer electrodes; then, the four piezoelectric cantilever beams can achieve a series connection by these inner and outer electrodes. Subsequently, theoretical and finite element models are established to analyze the effectiveness of the above structure. After fabricating the device, the measurement results demonstrate that the resonant frequency of the device is 7.24 kHz and the operating frequency is 56 Hz to 2360 Hz. At a frequency of 480 Hz, the sensitivity, minimum detectable acceleration, and resolution of the device are 2.448 mV/g, 1 mg, and 1 mg, respectively. The linearity of the accelerometer is good for accelerations less than 2 g. The proposed piezoelectric MEMS accelerometer has demonstrated high sensitivity and linearity, making it suitable for accurately detecting low-frequency vibrations.

## 1. Introduction

Microelectromechanical system (MEMS) accelerometers are the crucial inertial sensors that find extensive applications in fields such as inertial navigation [1,2,3,4], vibration measurement [5,6,7], medical diagnosis [8,9,10], health monitoring [11,12,13], and disaster warning [14,15,16]. Presently, there is a growing demand for MEMS accelerometers with small sizes, high sensitivity, and superior stability [17]. MEMS accelerometers can be classified based on their principles of operation, including capacitive [9,18], piezoresistive, resonant [19,20], and piezoelectric types [21,22,23,24]. Piezoelectric MEMS accelerometers exhibit several advantages over other types, including a wider operating frequency range, along with low power consumption, low-temperature dependence, and high sensitivity [25,26].

In recent years, many researchers have investigated the aspect of increasing the sensitivity of piezoelectric MEMS accelerometers. Gerfers et al. [27] presented the design of an accelerometer with a balanced bar structure that enhanced output sensitivity without requiring an increase in device size. The sensitivity of the device was experimentally determined to be 5.2 pC/g. Hui Zhou et al. [28] developed a piezoelectric MEMS accelerometer with the *d*_33_ mode based on a PZT piezoelectric film. The design of this accelerometer incorporated an interdigital transducer (IDT) electrode deposited on the cantilever beam to mitigate the impact of piezoelectric layer thickness on sensitivity, rather than the conventional sandwich structure. This approach resulted in an improved sensitivity of the accelerometer. The accelerometer demonstrated a voltage sensitivity of up to 4.55 mV/g. Shuzheng Shi et al. [29] designed a piezoelectric MEMS accelerometer with four L-shaped beam center proof mass based on a PZT piezoelectric film. The device incorporated a longer L-shaped beam, in contrast to the conventional straight beam structure, which increased the working area of the piezoelectric film and enhanced the sensitivity of the device, which reached 28.14 mV/g at 500 Hz. Yang et al. [17] presented a novel microelectromechanical system (MEMS) accelerometer designed using AlN piezoelectric material and polygonal topology. The device consisted of six topologies of electrodes that were connected in parallel, which resulted in an improved sensitivity of the device. The final sensitivity achieved by the device was 1.553 mV/g at 400 Hz. In summary, piezoelectric MEMS accelerometers are mainly used to improve sensitivity through the beam structure, operating mode, additional mass, and electrode connection.

Piezoelectric MEMS accelerometers generally use lead zirconate titanate (PZT), zinc oxide (ZnO), and aluminum nitride (AlN) as micromachined piezoelectric thin film materials [30]. AlN has gained significant interest due to its lead-free composition, low dielectric loss, low cost of fabrication, and compatibility with CMOS process technologies compared to ZnO and PZT. In Table 1, the main parameters of these three piezoelectric materials are compared. Despite its advantages, a drawback of using AlN films as a piezoelectric material is the lower piezoelectric coefficient. This lower coefficient can be a disadvantage for applications that require high sensitivity or large output signals.

In this paper, a piezoelectric MEMS accelerometer with high sensitivity is proposed. The sensitivity of the device is improved by using a ScAlN piezoelectric film, connecting the inner and outer electrodes of four cantilever beams in series and adding the proof mass. The addition of scandium (Sc) to AlN has emerged as a promising strategy to enhance the value of the piezoelectric coefficient [32]. The main structure of the device has a highly symmetrical four-beam structure with a single proof mass. Compared with single-cantilever beam and double-cantilever beam structures, a four-cantilever beam symmetrical structure has higher bandwidth and structural stability [33,34,35]. The top electrodes of the four beams are divided into inner and outer electrodes, which are connected in series to improve the voltage sensitivity. The size of the device is 2200 × 2200 μm^2^. Subsequently, theoretical and finite element models are established to analyze the effectiveness of the above structure. Then, the manufacturing process of the piezoelectric accelerometer is briefly introduced. Finally, the sensitivity, linearity, and resolution of the fabricated device are tested and discussed. The results show that the proposed accelerometer has great sensitivity and linearity.

## 2. Device Design and Simulation

### 2.1. Design

**Figure 1:**
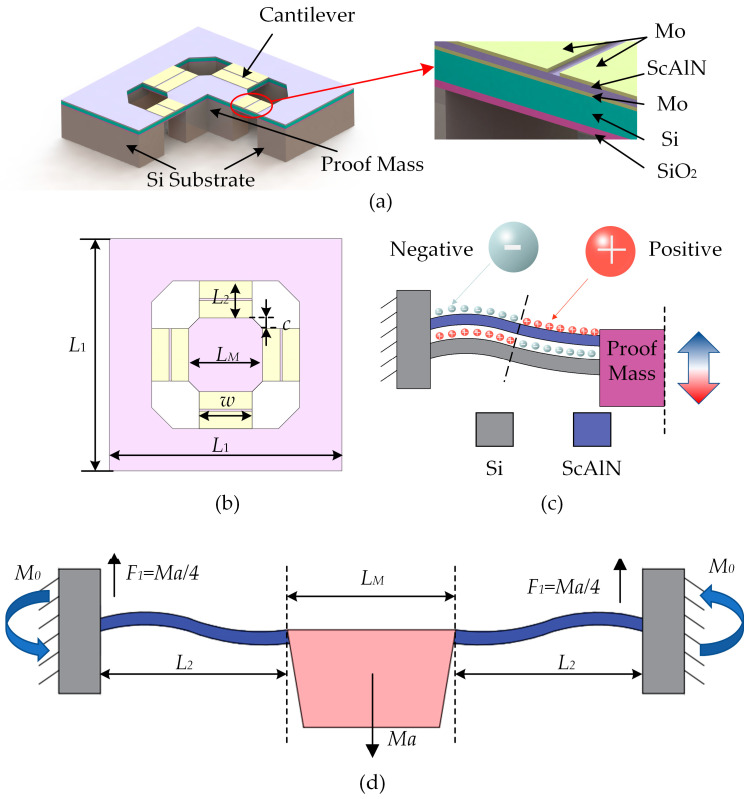
(**a**) Three-dimensional and (**b**) top view of the designed piezoelectric MEMS accelerometer. Schematic diagram of (**c**) the charge distribution and (**d**) the force on a single beam when Z-axis acceleration is applied.

The piezoelectric MEMS accelerometer is composed of three fundamental components: the cantilever beam, the proof mass, and the silicon substrate. The proof mass is supported by four beams. Figure 1a shows the three-dimensional model of the accelerometer. The cantilever beam is comprised of five layers of SiO_2_, Si, Mo, ScAlN, and Mo materials arranged from bottom to top. Figure 1b provides a top view of the device and exhibits chamfers on the proof mass and cavity edges to facilitate processing. The dimensional specifications of the device are presented in Table 2.

According to the principles of piezoelectricity and mechanical vibration, the piezoelectric MEMS accelerometer in this study is activated by an external force applied to the proof mass, which causes the cantilever beam to vibrate. The resulting vibration creates a change in polarization within the piezoelectric film of the cantilever beam, generating a voltage signal on the film’s surface. The cantilever beam exhibits opposite deformations in its two sections, resulting in a charge distribution that is illustrated in Figure 1c, where the red sphere represents a positive charge and the gray sphere represents a negative charge. To enhance the sensitivity of the piezoelectric accelerometer, the electrodes on the single-cantilever beam are divided into two parts, the inner and outer electrodes, and the device uses a series connection between the inner and outer electrodes to amplify the voltage output signal.

Structural analysis of the accelerometer reveals that, when subjected to acceleration “a” along the Z-axis, the central mass block undergoes vertical movement. Due to the highly symmetrical structure of the device, the four cantilever beams deform uniformly. As a result, the support reaction force at the root of the cantilever beam is distributed uniformly and is one-fourth of the force caused by the proof mass [29,33]. This phenomenon is shown in Figure 1d. Under the assumption that the mass of the beam is negligible and the bending of the proof mass can be treated as a point mass, we obtain Equation (1).

(1) $$EIw^{\prime \prime }(x)=F_{1}x-M_{0}$$

where *E* and *I* denote the Young’s modulus and extreme moment of inertia of the cantilever beam, respectively. The deflection curve of the cantilever beam is expressed as *w(x)*. The support reaction force at the fixed end of the cantilever beam is denoted by *F*_1_, *F*_1_ = *Ma*/4. Additionally, the limiting moment *M_0_* needs to be determined.

(2) $$EIw^{\prime }(x)=\frac{1}{2}F_{1}x^{2}-M_{0}x+H$$

(3) $$EIw(x)=\frac{1}{6}F_{1}x^{3}-\frac{1}{2}M_{0}x^{2}+Cx+J$$

where *H* and *J* are constants.

The deflection equation of the cantilever beam must satisfy the boundary condition, Equation (4):

(4) $$w\left(0\right)=0,w^{\prime }\left(0\right)=0,w^{\prime }\left(L_{1}\right)=0$$

In light of the fact that the beam of the accelerometer is a composite beam, comprising multiple layers of different materials, it is necessary to take into account the material properties and dimensions of each layer in the analysis of its deflection behavior under external forces.

(5) $$EI=\sum_{i}E_{i}\left(I_{i}+A_{i}Z_{i}{}^{2}\right)$$

(6) $$I_{i}=\frac{1}{12}w_{i}t_{i}{}^{3}$$

where *E_i_* and *t_i_* are the Young’s modulus and thickness of the *i*-th layer material, *A_i_* is the cross-sectional area of the *i*-th layer, *Z_i_* is the distance from the center of the *i*-th layer material to the neutral plane, and *w_i_* and *t_i_* are the width and thickness of the *i*-th layer in the multilayer cantilever beam.

Based on Equations (1)–(6), we are able to derive the following:

(7) $$M_{0}=\frac{1}{8}MaL_{2}$$

(8) $$w\left(x\right)=\frac{\left(\frac{1}{24}Max^{3}-\frac{1}{16}MaL_{2}x^{2}\right)}{\sum_{i}E_{i}\left(I_{i}+A_{i}Z_{i}{}^{2}\right)}$$

(9) $$M\left(x\right)=\frac{1}{4}Ma\left(x-\frac{1}{2}L_{2}\right)$$

Equation (9) reveals that the sign of the bending moment alters at the midpoint of the cantilever beam. This implies that the stress divides the beam into two parts at the middle point, and generates different charges at the internal and external ends of the beam, taking the center of the beam as the dividing point. These findings lend support to the previously suggested hypothesis.

(10) $$D=d_{31}\sigma$$

The formula for calculating the electrical displacement *D* of the piezoelectric accelerometer can be derived as shown in Equation (10). The parameter *d*_31_ represents the transverse piezoelectric coefficient of the piezoelectric film and *σ* denotes the normal stress in the X direction.

(11) $$\sigma =\frac{E_{p}Z_{p}}{R}=\frac{M\left(x\right)E_{p}Z_{p}}{\sum_{i}E_{i}\left(I_{i}+A_{i}Z_{i}{}^{2}\right)}$$

where *E_p_* is the Young’s modulus of the piezoelectric film, 1/*R* is the bending curvature of the cantilever beam when subjected to a bending moment *M(x)*, and *Z_p_* is the distance between the center of the piezoelectric layer and the neutral plane.

The neutral plane can be obtained by the following empirical formula:

(12) $$Z_{n}=\frac{\sum_{i}E_{i}t_{i}z_{i}}{\sum_{i}E_{i}t_{i}}$$

where *z_i_* is the distance from the intermediate axis of the *i*-layer material to the reference axis.

Therefore, the charge generated by a single electrode is:

(13) $$Q=\int Dw_{e}dl=\frac{d_{31}E_{p}Z_{P}M(x)\left(L_{M}+L_{2}\right)L_{2}}{2\sum_{i}E_{i}\left(\frac{1}{12}t_{i}{}^{3}+t_{i}Z_{i}{}^{2}\right)}$$

where *w_e_* and *dl* are the width and length of the electrode.

The two electrodes, which are separated by piezoelectric materials, can be considered as the upper and lower plates of a parallel plate capacitor. Therefore, the capacitance *C* can be determined using Equation (14).

(14) $$C=\varepsilon_{33}\frac{A}{d}=\frac{\varepsilon_{33}w_{e}L_{2}}{2t_{p}}$$

where *ε*_33_ is the dielectric constant of piezoelectric material. The voltage sensitivity of a single-cantilever beam connected in series with internal and external electrodes can be expressed as [36]:

(15) $$S=\frac{V}{a}=\frac{2Q}{aC}$$

The first resonant frequency of the device can be determined by using the Rayleigh–Ritz method [34,37].

(16) $$f=\frac{1}{2\pi }\sqrt{\frac{48\sum_{i}E_{i}(I_{i}+A_{i}Z_{i}{}^{2})}{M\cdot L_{2}{}^{3}}}$$

### 2.2. Acquisition of d_31_ of Sc_0.2_Al_0.8_N

The transverse piezoelectric coefficients *d*_31_ of piezoelectric membranes are fundamental to the design and simulation of piezoelectric devices. The cantilever beam technique has gained widespread acceptance as the predominant method for evaluating the transverse piezoelectric coefficient of piezoelectric thin films, primarily owing to its inherent advantages of ease of measurement and structural simplicity [38,39]. To obtain an accurate assessment of the transverse piezoelectric coefficient of ScAlN films integrated into piezoelectric devices, the present study employed the cantilever beam method founded on the inverse piezoelectric effect to conduct the measurements. In summary, the principle of the cantilever beam method is based on the inverse piezoelectric effect, where the application of a DC voltage to a piezoelectric cantilever beam results in the generation of stress within the piezoelectric layer, leading to a bending of the beam. The transverse piezoelectric coefficient *d*_31_ can be directly determined by measuring the tip displacement of the piezoelectric cantilever beam under varying DC voltage excitations and utilizing the equation that describes the relationship between the excitation voltage and displacement. The transverse piezoelectric coefficient *d*_31_ can be calculated by Equation (17) [40]:

(17) $$d_{31}=\frac{\left(E_{s}{}^{2}t_{s}{}^{4}+E_{s}E_{p}\left(4t_{s}{}^{3}t_{p}+6t_{s}{}^{2}t_{p}{}^{2}+4t_{s}t_{p}{}^{3}\right)+E_{p}{}^{2}t_{p}\right)c}{3t_{s}\left(t_{s}+t_{p}\right)E_{s}E_{p}l^{2}V}$$

where, in the given context, the parameters *E_s_*, *E_P_* and *t_s_*, *t_p_* represent the Young’s modulus and thickness of the support layer and piezoelectric layer, respectively. The length of the cantilever beam is denoted by *l*, while the voltage applied at both ends of the piezoelectric layer is represented by *V*. Additionally, *c* refers to the tip displacement of the cantilever beam.

**Figure 2:**
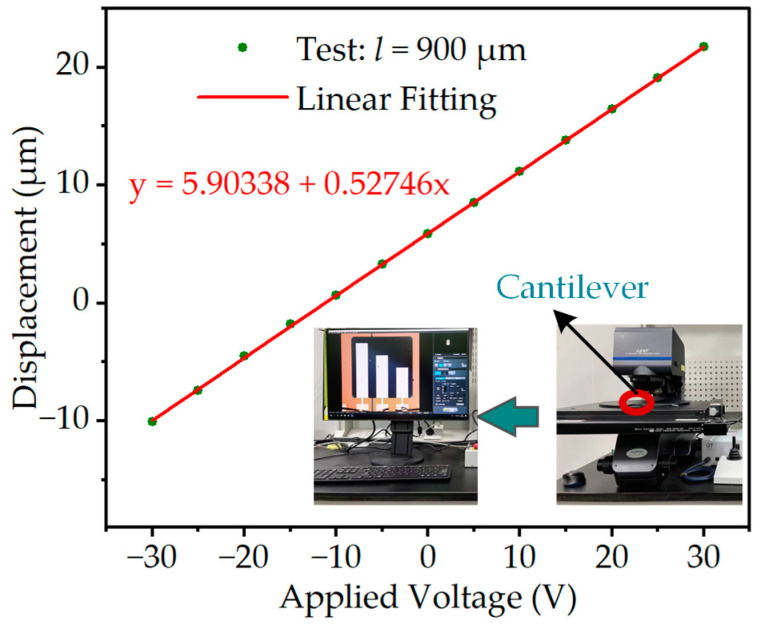
Cantilever beam method actual measurement results.

The experimental process involved the rigid connection of a piezoelectric cantilever beam, processed with the same technique as the MEMS accelerometer, to a PCB. The cantilever beam is electrically connected to the device by leading out gold wires using a wire-bonding machine and connecting the cantilever beam’s electrodes to the solder pads of the PCB via the gold wires. The PCB module is then placed in the measuring position of the confocal microscope (OLYMPUS OLS5000, Münster, Germany), as shown in Figure 2. Subsequently, a DC power supply is connected to the cantilever beam via wires, and a DC voltage is applied to the piezoelectric layer, ranging from −30 V to 30 V, with a step size of 5 V. The experimental results, which are presented in Figure 2, show that the cantilever beam exhibited initial bending due to residual stress generated during the processing. The length of the piezoelectric cantilever beam used in the experiment is 900 μm. By inputting the experimental data and main parameters from Table 3 into Equation (17), the *d*_31_ of the Sc_0.2_Al_0.8_N piezoelectric film is calculated to be −4.7661 pC/N.

### 2.3. Simulation

**Figure 3:**
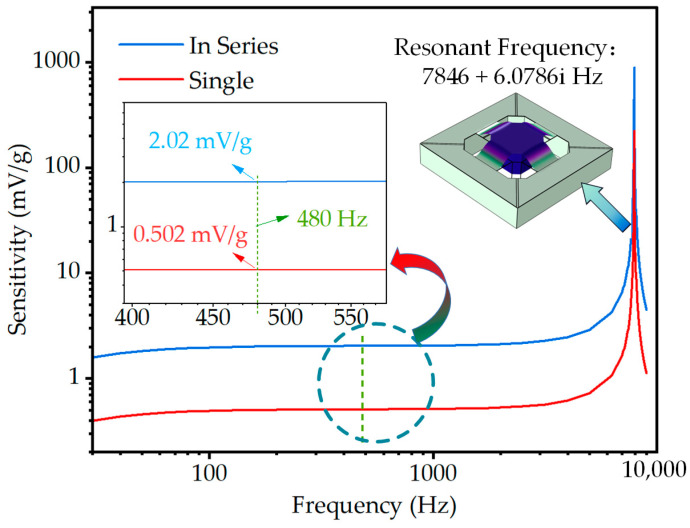
Voltage-sensitive response and first-order modal image from COMSOL finite element simulations.

In this study, the simulation of the resonance frequency and voltage sensitivity of the designed MEMS accelerometer is conducted using COMSOL Multiphysics 5.6. The first natural frequencies obtained from both theoretical calculations and simulations are 7766.7 Hz and 7846 Hz, respectively. These results demonstrate a high level of agreement between the theoretical model and the simulated device. Under 1 g acceleration excitation, the voltage sensitivity of the four-cantilever beams connected in a series the electrodes is found to be 2.02 mV/g at 480 Hz, whereas the voltage sensitivity of the single-ended cantilever beam inside and outside the electrodes is 0.502 mV/g, as shown in Figure 3. It is observed that the voltage sensitivity of the four-cantilever beams connected in series is higher than that of the single-ended cantilever beam, exhibiting a four-fold relationship. The simulation results are in good agreement with the theoretical calculations, indicating that it is feasible to increase the sensitivity of the sensor by connecting the inner and outer electrodes in series. The material parameters used in the simulation are shown in Table 3.

## 3. Fabrication and Characterization

**Figure 4:**
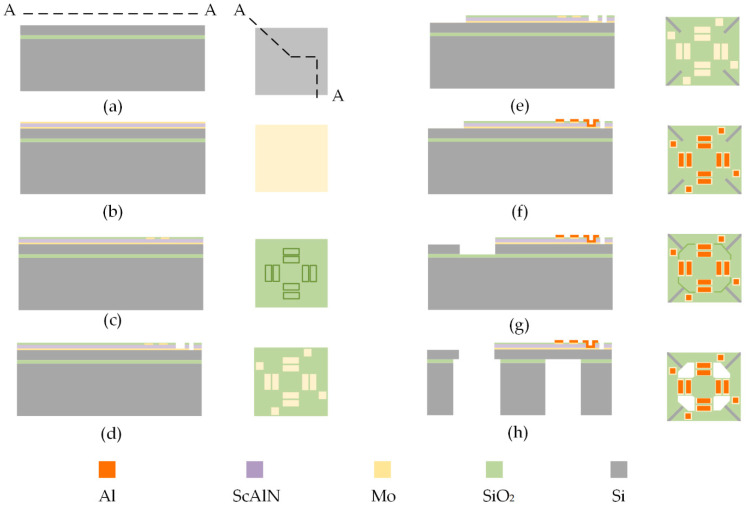
Cross-sectional view and top view of manufacturing process flow shown by section line A–A: (**a**) place SOI wafer; (**b**) depositing Mo electrode layer and ScAlN piezoelectric layer; (**c**) top electrode patterning. (**d**) top and bottom opening; (**e**) special structure etching; (**f**) Al pad patterning; (**g**) top releasing; (**h**) bottom releasing.

The present study details the fabrication process of a MEMS accelerometer on an SOI wafer, requiring six masks. The fabrication process is depicted through the A-A cross-sectional view and top view shown in Figure 4. Initially, an 8-inch SOI wafer is utilized as a silicon substrate, with a polished top silicon layer of 4 μm thickness, a buried oxygen layer of SiO_2_ of 1 μm thickness, and a bottom Si substrate of 400 μm thickness (Figure 4a). Magnetron sputtering is used to sequentially deposit a 0.2 μm thick Mo lower electrode layer, a 0.8 μm thick Sc_0.2_Al_0.8_N piezoelectric layer, and a 0.2 μm thick upper electrode layer on the polished top surface (Figure 4b). Dry etching is utilized by first patterning the top Mo electrode and the formation of an isolation layer by depositing SiO_2_ with a thickness of 0.2 μm (Figure 4c). To establish an electrical connection from the bottom electrode layer to the surface layer, followed by etching the Sc_0.2_Al_0.8_N piezoelectric layer and etching through-holes at the location connecting the top and bottom electrodes (Figure 4d). Excess SiO_2_, Sc_0.2_Al_0.8_N and Mo are etched to form a specific structure (Figure 4e). A 1 μm thick Al is deposited, etched, and patterned to align the electrodes and subsequent pads to enable electrical connection with the PCB (Figure 4f). Plasma etching is employed to remove the frontal Sc_0.2_Al_0.8_N piezoelectric layer, Mo electrode, and Si substrate to facilitate frontal etching (Figure 4g). Finally, a deep reactive-ion etching (DRIE) process is conducted to etch the Si structure layer from the back side of the SOI wafer, and buffered oxide etchant (BOE) of the buried oxygen layer is conducted to release the cantilever beam (Figure 4h).

**Figure 5:**
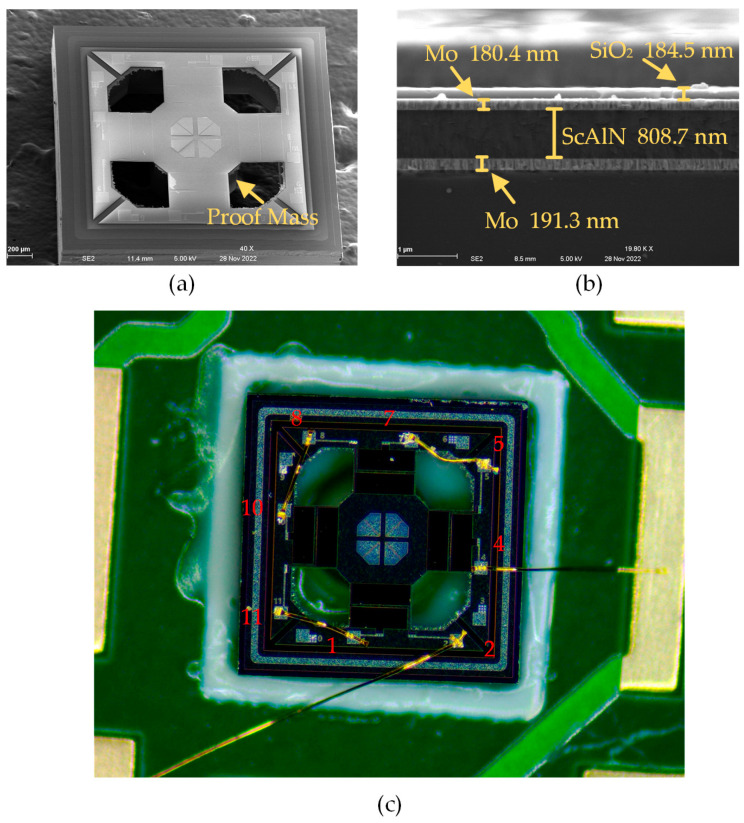
(**a**) Main view and (**b**) cross-sectional SEM images of the device. (**c**) Series connection diagram of the real device: inner electrodes 2, 5, 8, 11 and outer electrodes 1 4 7 10.

The MEMS accelerometer designed in this study is fabricated by Shanghai Industrial μTechnology Research Institute (SITRI). The structural characterization is presented in Figure 5a through scanning electron microscopy (SEM). The image demonstrates that there is a distinct height variation between the electrodes and the surrounding layer, indicating the electrodes’ favorable electrical connectivity. The physical dimensions of the device are consistent with the design values, as confirmed by direct measurement from the scale on the figure. Moreover, the proof mass in the center of the device is clearly visible when tilted.

The processed real object is analyzed by cross-sectional scanning electron microscopy. The resulting image is presented in Figure 5b. The image displays the layers of the object, which are arranged as follows, from top to bottom: a SiO_2_ protective layer of 184.5 nm, a Mo top electrode layer of 180.4 nm, a ScAlN piezoelectric layer of 808.7 nm, and a Mo bottom electrode layer of 191.3 nm. The thickness of each layer is slightly different from the design value, as the deviations in the deposition process during the processing lead to small discrepancies in the accelerometer performance characterization from the design value.

In Figure 5c, the MEMS piezoelectric accelerometer affixed to a printed circuit board (PCB) is shown. The designed MEMS accelerometer is securely bonded to the PCB board with an adhesive material. Furthermore, a cavity exists directly beneath the device on the PCB board to facilitate the unrestricted movement of the structure in the Z-axis direction. The device surface contains multiple pads, with labels corresponding to serial numbers 1, 4, 7, and 10, connected to the cantilever beam’s outer electrical stage. Additionally, the device surface contains pads with labels corresponding to serial numbers 2, 5, 8, and 11, which are connected to the cantilever beam’s inner electrode. The connectivity of the inner and outer electrodes of adjacent cantilever beams enables the series connection of the four cantilever beams in the accelerometer. The figure reveals the electrode connection sequence of the device, which follows the pattern of 4-5-7-8-10-11-1-2. Notably, number 2 and 4 pads are connected to the PCB pads through gold wires to enable subsequent performance characterization.

**Figure 6:**
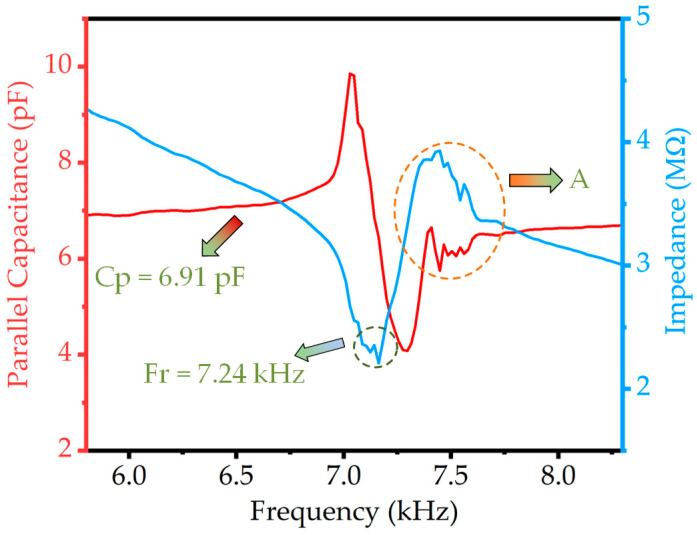
Impedance spectrum (blue) and parallel capacitance (red) of four cantilever beams with inner and outer electrodes in series.

**Figure 7:**
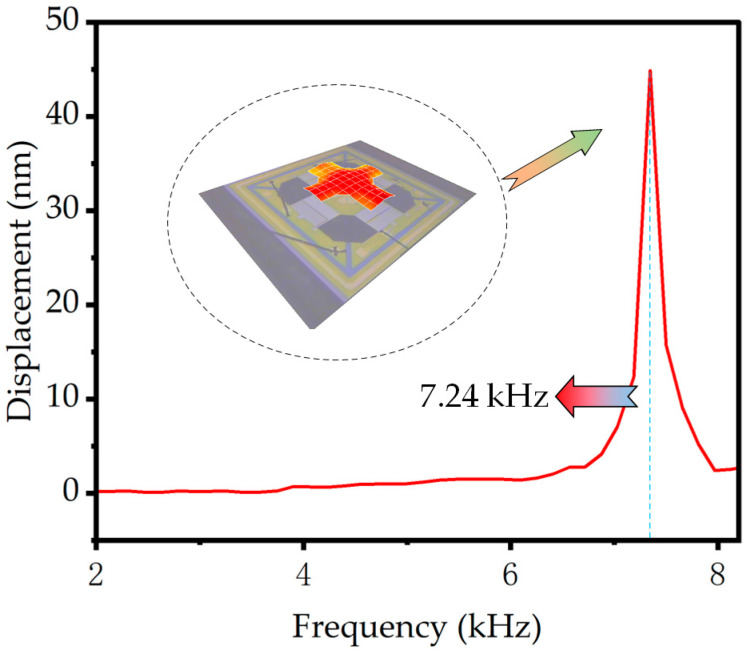
The frequency response of the proposed accelerometer is measured by LDV.

The impedance and static capacitances of the piezoelectric MEMS accelerometer are analyzed using an impedance analyzer (Keysight E4990A, Beijing, China). The measurement results of the device in the air are obtained when driven by a 100 mV peak-to-peak voltage, as shown in Figure 6. The resonant frequency of the device is found to be 7.24 kHz with a static capacitance of 6.91 pF. Furthermore, the orange dotted line encircling part A represents the distorted portion, which might attribute to the ringdown effect due to the inertia of the proof mass of the MEMS accelerometer causing significant displacement at the resonant frequency point. To further characterize the performance of the proposed MEMS accelerometer, an amplitude–frequency response and first-order mode analysis are carried out using a laser Doppler vibrometer (LDV, Polytec UHF-120, Irvine, CA, USA). Figure 7 depicts the device’s measurement results in air when driven by a 1 V peak-to-peak voltage. The piezoelectric MEMS accelerometer’s resonant frequency is found to be 7.24 kHz, with a point displacement of 42.7 nm at the resonant point. The resonant frequency of the device exhibits a discrepancy when compared to the simulation and theoretical calculation value, which is attributed to the presence of residual stress induced in the device during the fabrication process.

## 4. Results

**Figure 8:**
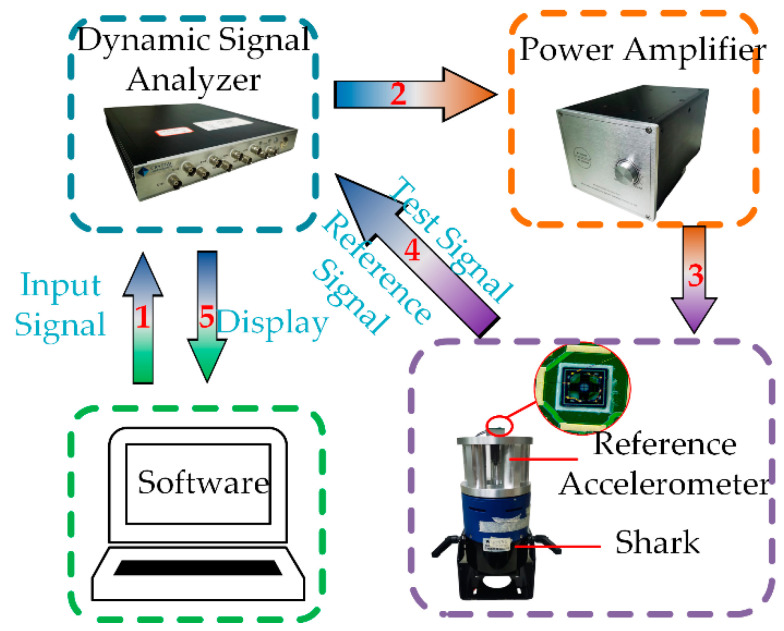
Experimental procedure for testing sensitivity.

The experimental study involving the calibration of the accelerometer is conducted using a measurement system depicted in Figure 8. The vibration testing apparatus comprises a single-axis shaker (PMG50, Metron Technology, Suzhou, China), power amplifier (DPM200A, Metron Technology, Suzhou, China), dynamic signal analyzer (SPIDER-80X, Metron Technology, Suzhou, China), and reference accelerometer (PCB-320C02). The accelerometer sensitivity characteristics are experimentally calibrated using the measurement system illustrated in Figure 8 over a frequency range spanning from 5 Hz to 5 kHz. Firstly, the SPIDER-80X is used to generate the excitation signal, which is then amplified by a power amplifier and transmitted to the shaker for excitation. Then, the MEMS accelerometer is fixed to the shaker, thereby subjecting the device to the same acceleration as the reference accelerometer connected back-to-back with the MEMS accelerometer. The reference accelerometer maintains the set acceleration value of the shaker output, resulting in a closed-loop controlled system. Finally, the accelerometer test signals are captured and transmitted to a PC for further performance analysis of the proposed sensor.

**Figure 9:**
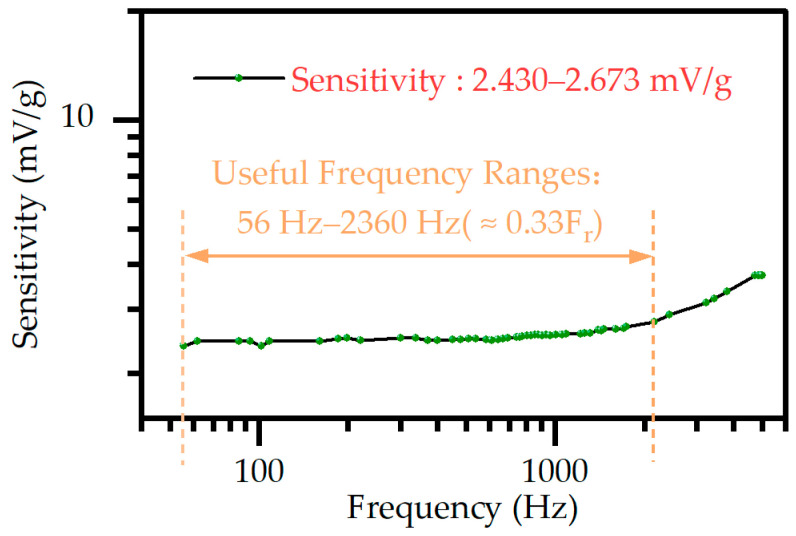
The frequency response curve of the accelerometer.

The measurement of sensitivity, linearity, operating frequency range, and minimum resolution are crucial to evaluating accelerometer performance. In this study, the frequency response curve of the proposed piezoelectric MEMS accelerometer is measured from 56 Hz to 5 kHz under 1 g acceleration excitation and is shown in Figure 9. Typically, the operating frequency range for piezoelectric MEMS accelerometers is limited to a sensitivity fluctuation of no more than 10% within this frequency range. As such, the maximum vibration frequency that can be accurately measured is roughly one-third of the resonant frequency of the accelerometer. The operating frequency is in the range of 56–2360 Hz and with a sensitivity range of 2.430–2.673 mV/g.

**Figure 10:**
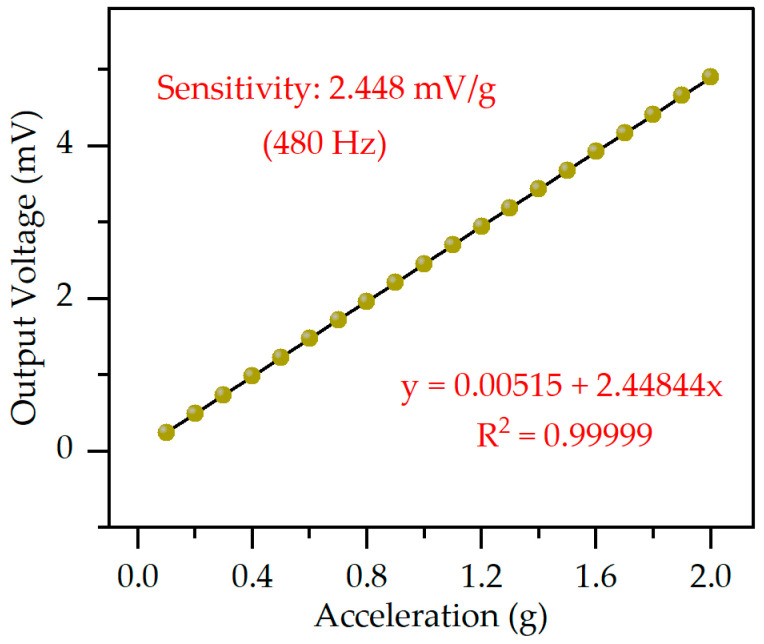
Excitation acceleration vs. output voltage to obtain sensitivity and linearity at 480 Hz.

Figure 10 illustrates the linearity of the proposed accelerometer in this study, which shows the relationship curve between the output voltage and acceleration values. The test is conducted by gradually increasing the acceleration excitation from 0.1 g to 2 g at a frequency of 480 Hz with a step size of 0.1 g. The voltage output is measured at each acceleration value for a period of 30 s. The sensitivity of the device is evaluated by calculating the slope of the linearly fitted curve, which is found to be 2.448 mV/g. The results indicate that the designed device exhibits good linearity over the range of 0.1 g to 2 g.

**Figure 11:**
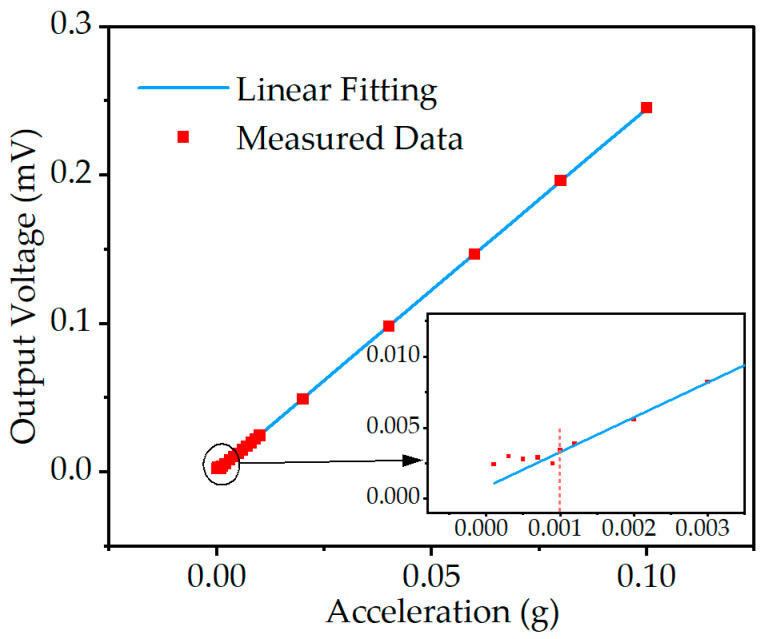
The minimum detectable acceleration of the accelerometer is proposed in this paper.

In the proposed accelerometer, at low vibration accelerations, the device’s output is no longer linear, which is shown in Figure 11. The minimum detectable acceleration of the accelerometer is 1 mg. For accelerations below this value, the output exhibits a significant deviation from linearity.

**Figure 12:**
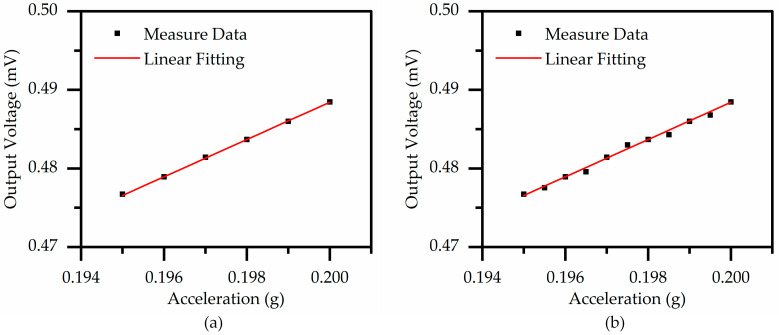
The output voltage of the piezoelectric MEMS accelerometer is found to have a relationship with acceleration at a frequency of 480 Hz. Sweep step size of excitation acceleration: (**a**) 1 mg; (**b**) 0.5 mg.

Figure 12 represents the response of the proposed accelerometer to acceleration at approximately 0.2 g and a frequency of 480 Hz under different scanning step sizes. The results of the study demonstrate that the proposed accelerometer is capable of detecting the change in acceleration at a minimum resolution of 1 mg, as depicted in Figure 12a, where the output voltage response displays good linearity. However, as illustrated in Figure 12b, when the scanning step size is reduced to 0.5 mg, the output response of the device becomes partially distorted, leading to imprecise measurements of the acceleration. Therefore, it can be inferred that the proposed accelerometer has a minimum resolution of 1 mg. In this study, the thermal noise of the proposed accelerometer is reported to be 85.6 nV/√Hz at 480 Hz and room temperature.

The piezoelectric MEMS accelerometer presented in this study demonstrates superior features including compact size and high sensitivity as compared to the results reported by other research groups (refer to Table 4). The voltage sensitivity of the device can be improved by connecting the inner and outer electrodes on the upper surface of the cantilever beam in series. These findings suggest that the proposed sensor can be utilized for accurate acceleration measurements and effectively used in low-frequency applications below 2.36 kHz.

## 5. Conclusions

This paper details the design, fabrication, and testing of ScAlN-based MEMS accelerometers with a trampoline structure produced using MEMS technology. The cantilever beam method is employed to measure the transverse sensitivity *d*_31_ of the Sc_0.2_Al_0.8_N film, yielding a value of −4.7661 pC/g, which is two to three times that of the pure AlN film *d*_31_. An acceleration of 1g is used to obtain the frequency response curve of the accelerometer. The resulting resonant frequency of the accelerometer with four cantilever beams and inner and outer electrodes in series is found to be 7.24 kHz, with a sensitivity of 2.448 mV/g at 480 Hz, operating in a bandwidth of 56 Hz to 2360 Hz. The proposed accelerometer exhibits good linearity at accelerations less than 2 g and has a minimum detected acceleration of 1 mg and a minimum resolution of 1 mg. The proposed piezoelectric accelerometer has the potential in high-sensitivity accelerometer applications.

## Figures and Tables

**Table 1:** Parameters of common piezoelectric materials [31].

Material	PZT	ZnO	AlN
Dielectric constant	300~1300	10.9	8.5~10.5
*d*_33_ (pC/N)	60~233	5.9~12.4	3.4~6.4
*d*_31_ (pC/N)	−40	−5.57	−0.98~−3.18
tanδ (10^5^ Vm^−1^)	0.01~0.03	0.01~0.1	0.003
Compatible with CMOS process	No	Yes	Yes

**Table 2:** The structure parameters of the proposed MEMS accelerometer.

Parameters	Physical Descriptions	Values (μm)
*L*_1_	Length of the whole device	2200
*L*_2_	Length of the single-cantilever beam	350
*L*_M_	Length of the proof mass	700
*C*	Length of the chamfered edge of the proof mass	100
*W*	Width of the single-cantilever beam	350
*t_so_*	Thickness of the SiO_2_ layer	1
*t_s_*	Thickness of the Si layer	4
*t_p_*	Thickness of the Sc_0.2_Al_0.8_N layer	0.8
*t_e_*	Thickness of the Mo layer	0.2
*t_m_*	Thickness of the proof mass	400

**Table 3:** Main parameters of the materials used in the simulation [25,26,41].

Materials	Young’s Modulus (GPa)	Poisson’s Ratio	Density (kg/m^3^)	Relative Permittivity
Si	130	0.28	2329	-
Mo	312	0.31	10,200	-
Sc_0.2_Al_0.8_N	230	0.31	3318	13.7
SiO_2_	70	0.17	2200	-

**Table 4:** Comparison of the main parameters of accelerometers.

Author	Chen, Z.-H. et al. [42]	Hu, B. et al. [25]	Yang, C. et al. [17]	This Work
Materials	AlN	AlN/ScAlN	AlN	ScAlN
Sensitivity (mV/g)	1.49	7.95	1.533	2.448
Resonance frequency (kHz)	7.2	1.29	98	7.24
Device structure	Annular	Trapezoidal-with-corners-shaped cantilever	Polygon topological cantilevers	Trampoline
Moving part size (mm^2^)	63.62	14.25	0.723	0.97

## Data Availability

Not applicable.
